# Isolation, Characterization, Cryopreservation of Human Amniotic Stem Cells and Differentiation to Osteogenic and Adipogenic Cells

**DOI:** 10.1371/journal.pone.0158281

**Published:** 2016-07-19

**Authors:** Shiva Gholizadeh-Ghaleh Aziz, Fatima Pashaei-Asl, Zahra Fardyazar, Maryam Pashaiasl

**Affiliations:** 1 Women’s Reproductive Health Research Center, Tabriz University of Medical Sciences, Tabriz, Iran; 2 Molecular Biology Laboratory, Biotechnology Research Center, Tabriz University of Medical Sciences, Tabriz, Iran; Flinders University, AUSTRALIA

## Abstract

Human stem cells and progenitor cells can be used to treat cancer and replace dysfunctional cells within a tissue or organ. The objective of this study was to identify the appropriate cells type in regenerative medicine and targeted therapy. As an alternative to embryonic and bone marrow stem cells, we examined human amniotic fluid stem cells (hAFSCs), one of the potential source of multipotent stem cells isolated from both cell pellet (using single-stage method), and supernatant of human amniotic fluid. Source of isolation and unique property of the cells emphasize that these cells are one of the promising new tools in therapeutic field. Double sources for isolation and availability of the left over samples in diagnostic laboratory at the same time have less legal and ethical concerns compared with embryonic stem cell studies. Cells were isolated, cultured for 18^th^ passage for 6 months and characterized using qPCR and flow cytometry. Cells showed good proliferative ability in culture condition. The cells successfully differentiated into the adipogenic and osteogenic lineages. Based on these findings, amniotic fluid can be considered as an appropriate and convenient source of human amniotic fluid stem cells. These cells provide potential tools for therapeutic applications in the field of regenerative medicine. To get a better understanding of crosstalk between Oct4/NANOG with osteogenesis and adipogenesis, we used network analysis based on Common Targets algorithm and Common Regulators algorithm as well as subnetwork discovery based on gene set enrichment. Network analysis highlighted the possible role of MIR 302A and MIR let-7g. We demonstrated the high expression of MIR 302A and low expression of MIR let7g in hAFSCs by qPCR.

## Introduction

Over the past two decades, a great interest has been paid to stem cell therapy in cancer therapy [1], regenerative medicine [2] and other applications [3]. Three main classifications of stem cells are embryonic, adult and fetal stem cells which first two have attracted many of researchers in the field of biology; however fetal stem cells need more attention and elucidation which is our research focuses. Embryonic stem cells (ESCs) can easily derived from blastocysts [4, 5] and hold ability of forming aggregates (embryoid bodies) producing a variety of specialized cells including cardiac [6], neural [7] and pancreatic cells [8] and so on, but ethical issues and their potential ability to initiate teratoma may eventually prohibit their usefulness clinical application [9, 10]. On the other hand, adult stem cells are multipotent and available in small numbers in almost all tissues to fulfill cell homeostasis in natural aging or repair tissue as a result of injury or diseases. Multipotent autologous stem cells are isolated from a number of tissues such as adipose tissue as well as neural [11], reproductive [12], cardiac [13], olfactory [14], endothelial [15] and digestive system [16, 17].

Although autologous types of stem cells have some advantages and are not subjected to issues but the main barriers could be rare in the number and difficulty of isolation, purification and maintenance to reach the required number for transplantation. In order to avoid these problems and overcome to limitations, scientists have looked to other sources for pluripotent cells such as amniotic fluid stem cells. Amniotic fluid is well-known in diagnostic fields and comprise multiple cell types derived from the developing fetus [18, 19] as well as are safe and reliable screening tool for genetic and congenital diseases in the fetus [20]. Cells within this heterogeneous population are able to give rise to various differentiated cells including adipose, osteoblasts, muscle, bone and neuronal lineages [20–23].

Human amniotic fluid stem cells (hAFSCs) possess many characteristics, which may identical to human ESCs, such as: expression of embryonic markers, the ability to maintain their telomeric length, potential to give rise to multi-lineage and capable to maintain in culture for many population doublings [24].

When they are stimulated with different growth factors, molecules and medium, have potential to give rise to multiple cells, derived from all the three germ layers [25, 26]. They appear to be safer and more pluripotent than stem cells derived from bone marrow [24]. Unlike ESCs, hAFSCs cells do not form tumors or teratoma in vivo. A low risk of tumorigenicity would be advantageous for future therapeutic applications [27]

There are two main methods for isolation of hAFSCs [22–28] which we applied for isolation while other studies have been used only one of them. The multipotency of hAFSCs are analyzed using RT-PCR, qPCR and flow cytometry and in our study we focused on two mesenchymal stem cell surface markers of hAFSCs (CD44 and CD90) and two hematopoietic stem cell surface markers (CD 31, CD 45). In this study, we looked at the potential of hAFSCs to give rise to adipogenic and osteogenic lineages using histochemical staining. Furthermore, we assessed cryopreservation of hAFSCs, in which optimum condition was established for cryopreservation of hAFSCs. So, we provide a cell bank of isolated and characterized hAFSCs, which in future may provide a convenient source to patients as therapeutics applications.

We used network analysis based on algorithms including Common Targets, Common Regulators, and Gene Set Enrichment to obtain a better view on the relationships between Oct-4 and NANOG with adipogenesis and osteogenesis.

## Methods and Materials

### Sampling

Five milliliters of human amniotic fluid samples were obtained from 10 patients undergoing amniocentesis for routine karyotype screening and evaluated in Al-Zahra hospital (Tabriz, Iran). Amniotic fluid samples were donated by mothers for this research after clarification and filling patient consent forms under the ethic guide line of Tabriz University of Medical Sciences. All participants provide their written informed consent to participate in this study; the ethics committee approves this consent procedure: (registered number 5.4.753 at ethic committee of TUMA). The Amniocentesis was performed under gynecologist supervision by sonographer guides using a 22G Needle.

### Media and Cell Culture

Amniotic cells were isolated from human amniotic fluid through centrifuging samples at 450 g for 10 min and preserved in 37°C prior to prepare culture condition. To optimize the best culture condition for cells, we used two types of media. AmnioMAX II Complete Medium (Gibco, cat# 11269), this medium is developed for the short term culture of human amniotic fluid cells and is premixed complete media. Type two media was DMEM- L ( Dulbecco's Modified Eagle Medium Low Glucose) supplemented with 15% FBS (Fetal Bovine serum), 10 ng/ml basic fibroblast growth factor (bFGF (, 1% glutamine and 1% penicillin/streptomycin all supplied by (Gibco).

The resulting cell pellets were homogenized in both culture media separately and were seeded in 6 wells plates with defined medium (AmnioMAX or DMEM-L) and incubated at 37°C in a humidified gas environment of 5% CO2 in air. After one week of culture, there were no attached cells in plates containing DMEM-L media but cells were divided into two sets in AmnioMAX medium:

1. Some cells which attached to the plate were kept in this plate for 3 weeks and cultured based on Single-stage method [28]
2. Other Cells which were detached and floating in media were collected and cultured based on Two-stage method. So the supernatant was collected, transferred and cultured in new plate (P0) [22].

After 3 weeks, the cells of both methods showed multilayers morphology, which had the 90% confluency in AmnioMAX medium. The medium were replaced two times per week. After 3 weeks, primary passage (P1) was performed through harvesting cells by trypsinization. Cells were transferred to T25 flasks containing type two media (Gibco) and incubated at 37°C with 5% CO2 atmosphere for expansion. Cells were able to reached 80–90% confluence in 5 to 7 days which were passaged. The putative hAFSCs were cultured for 18^th^ passages during 6 months.

### Characterization of Putative HAFSCs Lines

The goal of this experiment was to characterize established putative cell lines (randomly 4 of 10 samples) at 3th-5th passage after extended culture for pluripotency markers using RT-PCR and mesenchymal stem cells markers using FACS analysis.

### Characterization of Pluripotency

Pluripotency statuses of cell lines were evaluated at P3-5 before cryopreservation. Cells were checked by reverse transcription polymerase chain reaction (RT-PCR) for gene expression of pluripotent markers including Oct4 and NANOG which were visualized by gel electrophoresis. In addition, cells were prepared for FACS analysis to assess for mesenchymal stem cells markers such as CD90 and CD44 and hematopoietic markers such as CD31 and CD45.

### Total RNA Extraction, cDNA Synthesis and RT-PCR

Total RNA was extracted from 5–6×10^5^ undifferentiated hAFSCs from 3th-5th passages, using RNX-Plus kit (CinnaGen, cat# RN7713C) according to the manufacturer’s protocol. Complementary DNA (cDNA) was synthesized from total RNA using Termo Scientific Revert Aid First Strand cDNA Synthesis Kit (Fermentas, cat# K1621) with oligo-dT primer in a 20 μl reaction mixture which was performed in a Thermal Cycler (PeQLab). Primers used for amplification are listed in Table 1. All samples were checked for b-actin (housekeeping gene) as an internal control to verify the achievement of the RT reaction and then other pluripotency genes were checked with its specific primers. RT-PCR using the Hyper Script RT master mix (GenAll, cat# 601–710) was performed with 2μl of the single stranded cDNA sample for detection of NANOG and Oct4 (POU5F1) gene expression. After initial denaturation at 94°C for 4 min, PCR amplification was carried out at 94°C for 30 sec, followed by annealing temperature at 56°C for 30 sec, and 72°C for 30 sec and extension at 72°C for 5 min for a total of 35 cycles. Amplified PCR products were separated on a 2% agarose gel by electrophoresis and the bands were visualized by dying buffer and photographed with a gel doc.

**Table 1:** Primers used for RT-PCR.

Gene name	Primer forward (F) and revers (R)	PCR size
Oct4 (POU5F1)	F 5' CCATGCATTCAAACTGAGGT 3'	146bp
	R 5' CCTTTGTGTTCCCAATTCCT 3'	
NANOG	F 5' AGTCCCAAAGGCAAACAACCCACTTC 3'	161bp
	R 5' TGCTGGAGGCTGAGGTATTTCTGTCTC3'	
Beta actin	F 5' GGCACCCAGCACAATGAAGA 3'	342bp
	R 5' CGACTGCTGTCACCTTCACC 3'	

### Quantitative Real Time PCR (qPCR)

RNA extractions and cDNA syntheses were carried out as described above, mRNA levels of β-Actin, NANOG and Oct4 genes were assessed in samples isolated from P5 (early P) and P7 (late P). QRT-PCR reactions were carried out using SYBR® Premix Ex Taq™ II (Tli RNase H Plus) (Cat # RR820) in a Rotor-Gene 6000 Real-time PCR Detection System (Corbett, UK), according to the manufacturer’s instructions. PCR amplification has conducted with the following settings: hold at 95°C for 5 minutes, followed by 40 cycles: denaturation for 15 seconds at 95°C, annealing for 35 seconds at 60°C, and extension for 15 seconds at 72°C. Relative gene expression was calculated with the 2^–ΔΔCt^ method, the amount of target, normalized to an endogenous reference and relative to a calibrator, where ΔCt = Ct target gene–Ct endogenous reference and ΔΔCt = ΔCt sample– ΔCt calibrator. All tests have performed in triplicate [29]. Primer sequences for target genes were[30]: OCT4 forward primer: 5´-CCATGCATTCAAACTGAGGTG-3´; OCT4 reverse primer: 5´-CCTTTGTGTTCCCAATTCCTTC-3´; NANOG forward primer: 5´-AGTCCCAAAGGCAAACAACCCACTTC-3´; NANOG reverse primer: 5´-TGCTGGAGGCTGAGGTATTTCTGTCTC -3´, β-Actin forward primer: 5´-CCTTCCTTCCTGGGCATG-3´; β-Actin reverse primer: 5´- TCCTGTCGGCAATGCCAG -3´ [31].

### Flow cytometric analysis

The cells (passage 3–5) were detached with trypsin and washed twice in FACS wash (PBS+ 2% FBS). Then, the cells were centrifuged in 1500 rpm for 3 minutes. The resulting cell pellet was resuspended and filtered out using a 40 μm cell strainer. Then the cells were incubated with a 1/30 dilution of fluorescein-conjugated antibody. Then, cells were diluted in 100μl of FACS wash containing 1/1000 concentration of PI (propidium iodide) in order to exclude dead cells and analyzed by flow cytometry with a BD FACS Calibur Flow cytometer (Ref: 342976) at total of 10000 events for each cell line. We evaluated CD90 (BD pharmigen, cat# 560977), CD 44 (BD pharmigen, cat# 555596), mesenchymal stem cell markers, and CD31 (PECAM-1, cat# FAB3567c) and CD 45(abcam, cat# ab65952) as hematopoietic markers.

### Cryopreservation of hAFSCs

Pluripotent cell lines were frozen[32] with some modifications: briefly; following trypsinizing, the cells were harvested and centrifuge at RT for 3 min at 400g, the supernatant was discarded and the cell lumps were resuspended, counted by haemocytometer and 1×10^6^ cells were prepared per cryovial (Nunc, Thermo Fisher Scientific). Cryovial containing each Freezing medium (10% dimethylsulfoxide (DMSO; Sigma, St Louis, MO, USA), 90% FBS) after the addition of the cells were transferred to -20°C for 45 minutes (optional) and then to -70°C for 24h. All samples were then stored in a liquid nitrogen tank for storage and further analysis.

To thaw cryopreserved cells, cryovials were rapidly dipped in a water bath set at 37°C. Then the cells were transferred to a 15mL falcon tube and 10mL amniotic fluid stem cells (AFSCs) medium was gradually added. The supernatant was discarded after centrifugation at RT for 3 min at 400g. The o pellet was resuspended in AFSCs medium and the numbers of cells were calculated using Trypan blue. The two methods were compared for colony morphology and cell survival following thawing.

### Doubling Time of AFSCs

Well growing cells from passages 4,7 and 10 were harvested by trypsinization, resuspended in media, plated into 6-well plates at 60000 cells per well, and incubated for 24, 48, 72 and 96 hours in a humidified 37°C incubator under 5% CO2. Then, the viability of cells was assessed and the mean values were used to plot cells growth curves. The population doubling time was calculated according to the following formula [33]. Like previous step in this process was repeated three times, and statistical analysis was performed.

$$Doubling\;time\;=\frac{lg2}{\left(\text{lg}\;ultimate\;cell\;number-\text{lg}\;primary\;cellnumber\times termination\;incubation\;time\right)}$$

### Osteogenic and Adipogenic Induction

HAFSCs were maintained in osteogenic and adipogenic induction media 3 weeks. Osteogenic induction media consisted of α-MEM supplemented with 10% FBS, 0.1 μmol/l dexamethasone, 10mmol/l β-glycerol phosphate, 50μmol/l ascorbate (Gibco). Adipogenic induction media contained α-MEM supplemented with 10% FBS, 1μmol/l dexamethasone, 5μg/ml insulin, 0.5 mmol/l isobutylmethylxanthine and 60μmol/l indomethacin (Gibco). Alizarin red and Oil Red was utilized to identify osteogenic and adipogenic differentiation, respectively.

## Statistical analysis

Statistical analysis was performed with SPSS 14 software. ANOVA (Tukey HSD), 2 Sample T-test and Post Hoc test were used to compare groups. Data are represented Mean ± Standard deviation. The differences were considered significant when P<0.05.

### Network Based Analysis of Crosstalk between Oct-4 and NANOG

Using Oct-4, NANOG osteogenesis, and adipogenesis as input entities, we used ResNet an enriched text-mining based of mammalian database of gene/protein/small RNA interactions, implemented in the Pathway Studio 11.0.5 (Elsevier) [34]. ResNet is enriched with different interaction sets by Medscan language programming including regulation, expression, regulation, promoter binding, transport, protein binding and modification, molecular synthesis, chemical reaction, miRNA effect, and small molecule function [35].

Three algorithms were used in network construction including Common targets, Common regulators as well as upstream and downstream subnetwork discovery based on Gene Set Enrichment (GSE) approach at p = 0.01 [36–39]. Common target algorithm determines which targets/mechanisms will be activate/inactivate by alteration of Oct-4 and NANOG in differentiation to osteogenic and adipogenic cells. In other words, common target tries to find the goal/consequence of modulation of gene/proteins. At first, target connectivity was set to 2 to find downstream targets of NANOG and Oct-4. Then, cellular processes of adipogenesis and osteogenesis were added to Common Targets network. The entities were kept at the final network which were targets NANOG and Oct-4 and had also interactions with adipogenesis and osteogenesis.

In contrast, common regulators algorithm identifies the regulators which have the highest number of regulation/expression relationships with the altered proteins by Oct-4 and NANOG. In other words, common regulator algorithm tries to find the managers/commanders/regulators of the altered proteins in proteomics data.

As previously described [40–44], upstream subnetwork discovery demonstrates the subnetworks of targets/components which are under the control of a particular subnetwork of the altered genes. Reversely, downstream subnetwork discovery unravels the significant regulatory subnetworks within the regulatory subnetworks with high chance/probability of generation during differentiation at p = 0.01.

The underlying references (papers) of relationships and their Medline and PubMed IDs were obtained. The cellular location and type of different proteins in this network were determined based on Cellular Component definition from Gene Ontology Consortium (http://geneontology.org/) implemented in Pathway Studio package as well as Comparative GO web tool [45, 46].

### QPCR analysis of microRNAs

Total RNAs was extracted according to the above mentioned protocol. Total RNA is briefly exposed to RNAase-free DNAase I. BonMiR kit was used for qPCR analysis of microRNAs. First, Bon-miRBonmiR 1st-strand cDNA synthesis kit (Cat# BON209001) was used to elongate miRNAs in a polyadenylation reaction; following polyadenylation, the RNA was used as template to produce 1st-strand cDNA. MicroRNA Reverse transcription (RT) was carried out using miRNA specific stem–loop primers (BonMiR). RT reactions with a final volume of 20 μL and following primers were performed: MIR 21 F: “ACG TGT TAGCTT ATC AGA CTG A”, MIR 302A F: “CCG CTA AGT GCT TCC ATG TTT TG”, MIR 137 F: “CGT TAT TGG TTA AGA ATA CGC” and MIR Let7g F: “CGA TGA GGT AGT AGT TTC TAC AGT”. Snord primers, universal reverse primer: GAG CAG GGT CCG AGG T and forward primer: ATC ACT GTA AAA CCG TTC CA were used as an internal control to adjust the changes in the amount of cDNA in various samples. As Negative control, human fibroblast cell cline was used.

The cDNA synthesis reaction was performed according BONmiR miRNA 1st-Strand cDNA Synthesis Kit; Cat# BON209001. In brief, 1.0 μl RT enzyme, 10 μM Bon-RT primer, 100 mM dNTP mix, 10 μl polyadenylated and DNase treated total RNA, 2.0 μl 10× RT buffer were used and final volume was reached to 20 μl with RNase-free water to bring were used and thermocycler program was as follows: 55⁰ C for 5 min, 25⁰ C for 15 min, 42⁰ C for 30 min, and 95⁰ C for 5 min.

MiRNAs were measured by qPCR using BONmiR High-Specificity miRNA qPCR Core Reagent Kit (Cat# BON209002) and individual specific miRNA primers. Universal reverse primer (available in BonMiR QRT-PCR kit) and forward primer were used. Each reaction (20 μL) was performed in triplicate. Real time PCR was performed using the following conditions: 95°C for 20 sec, followed by 40 cycles of 95°C for 5 sec and 60°C for 30 sec.

We used 2^-ΔΔCt^ method to evaluate expression levels of MIR 21, MIR 302A, MIR 137, and MIR let-7g for all samples. Comparison of miRNA expression values carried out using 2-sample T-test.

## Results

### Isolation Using Single-Stage & Two-Stage Methods

In this study, we successfully isolated human amniotic stem cells in both, supernatant of initial culture (Two-stage method) and initial pellet of cells (Single-stage method), but only in AminoMAX media. Then, cell bank were prepared with optimum condition. After one week of first culture in AminoMAX, all samples were analyzed using invert microscopy and results showed the excessive cell growth in size and numbers. The Multilayer growths of cells were appeared after 3 week of first culture. In this condition, the confluency of cells was up to 90%. In other hand, in DMEM Low media, the cells were not able to adhere to plate and this media is not suitable for isolating AFSCs in P0. In P0 AminoMAX media was the optimum media for isolation of human amniotic stem cells.

### Morphological Characterization

**Fig 1:**
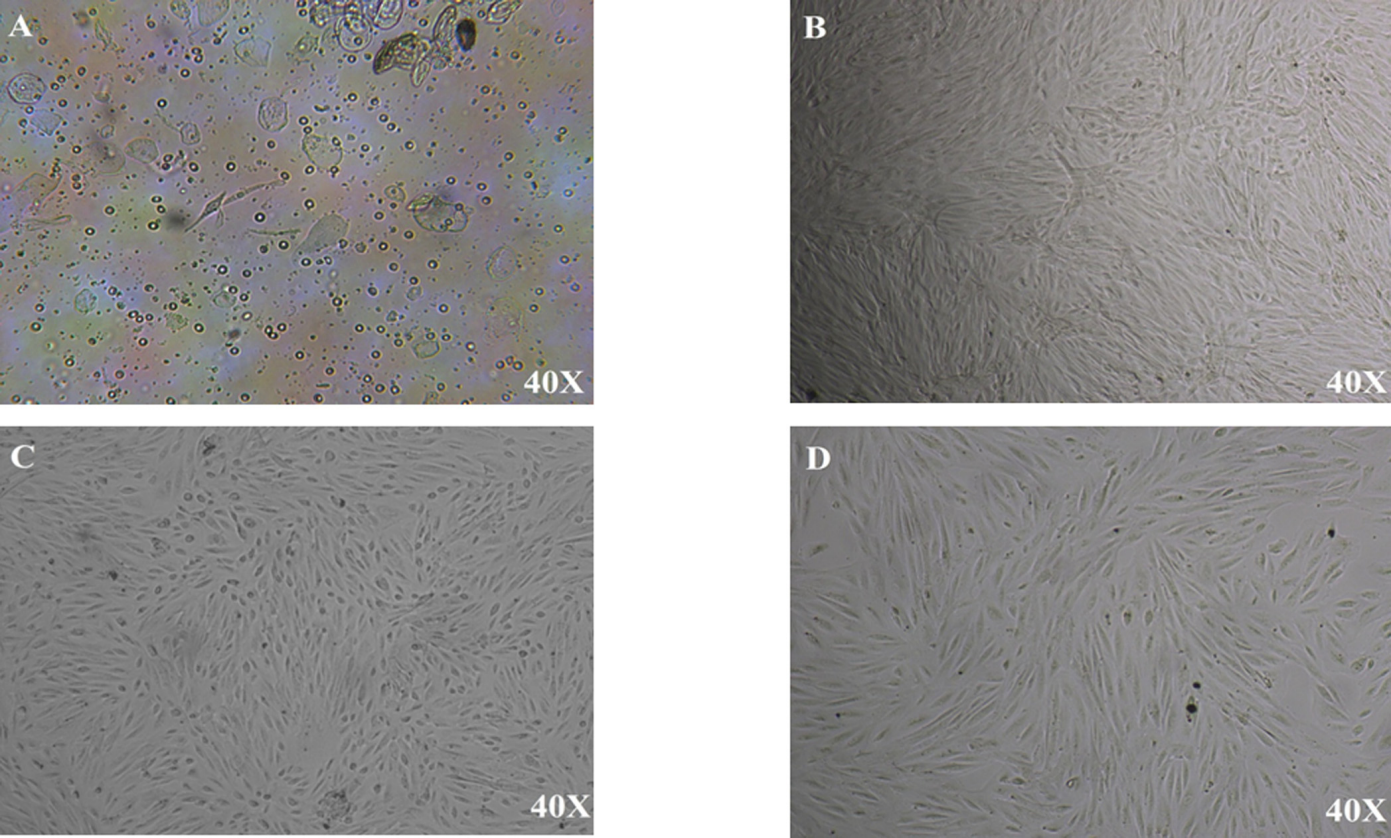
(40x magnification was used) Fig A. Primary passage of DMEM-L. Cells have not ability to adhere to plate. Fig B. Primary passage of AmnioMAX. The Multilayer growths of cells were appeared after 3 weeks of first culture. Fig C. Cells in primary passage those were isolated from supernatant. Fig D. Passage 1 the epithelioid population rapidly disappeared from culture and AFSCs showed a fibroblast-like morphology

In P0, the morphology of cells was spindle-shaped and polygonal and cells aggregated in colony form. In other hand, in passage1 (P1), the epithelioid population rapidly disappeared from culture and the clones showed a fibroblast-like morphology. (Fig 1- 40x magnification was used).

### Total RNA extraction, cDNA synthesis and RT-PCR

**Fig 2:**
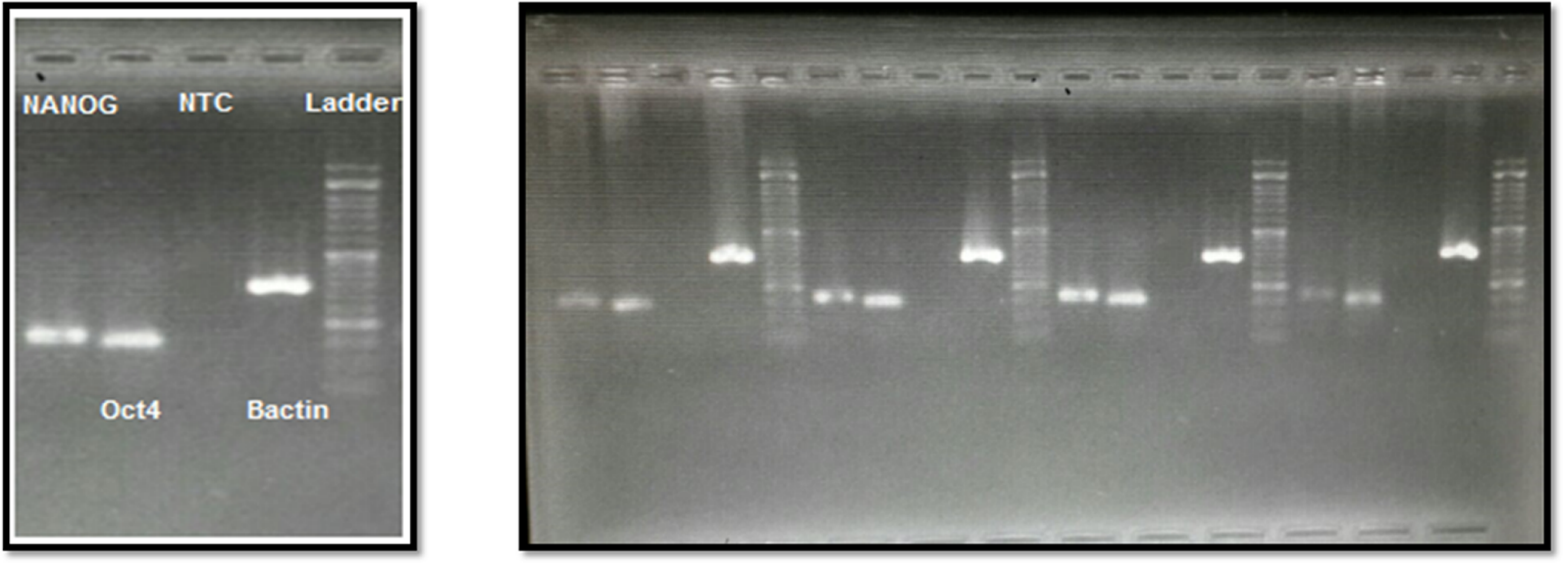
**(A, B).** Gene expression profile of amnion-derived stem cells.**A**; Sample 2, Ladder, Bactin, Negative control, Oct4, NANOG. **B**; For each 4 samples from right to left; S1, 2, 3, 4 were showed on gel electrophoresis

All samples between 3^th^ -5^th^ passages analyzed by RT-PCR showed mRNA expression of stem cell markers (pluripotency markers) such as Oct-4 (POU5F1), and NANOG, detectable relative to the housekeeping gene b-actin (Fig 2).

### Real Time PCR (qPCR)

**Fig 3:**
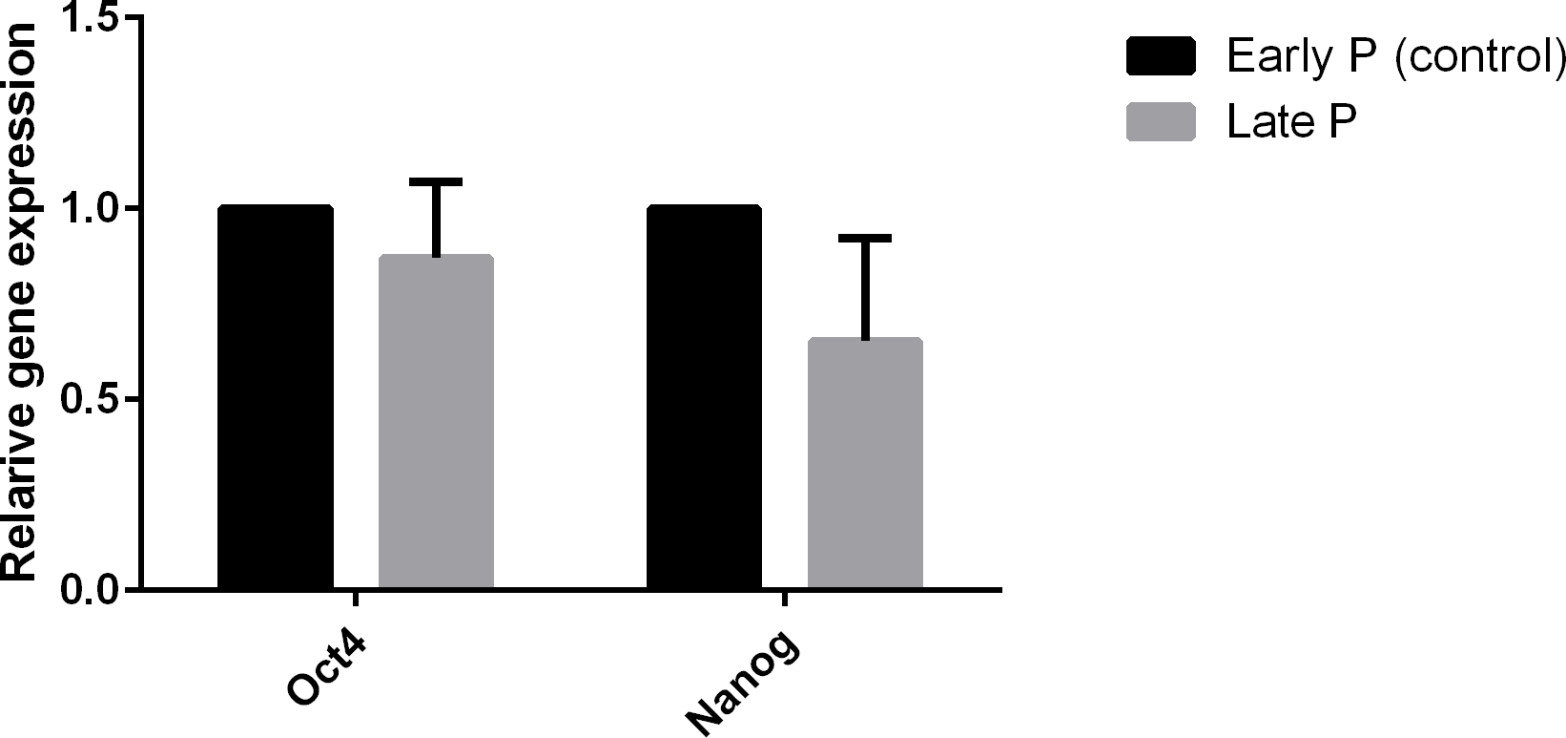
Relative expression of Oct4 and NANOG at early and late passages. Expression level of Oct4 and NANOG were high at P5 (early passage) compared with P7 (late passage) (P< 0.05).

The expression of β-Actin, OCT4 and NANOG were further examined by quantitative relative real-time PCR. mRNA expression of both samples (early and late passages) showed that AFSCs are a source of pluripotent stem cell markers of Oct4 and NANOG; however expression level of Oct4 and NANOG was high at P5 (early passage) compared with P7 (late passage) (P< 0.05) (Fig 3).

### Flow Cytometry

**Fig 4:**
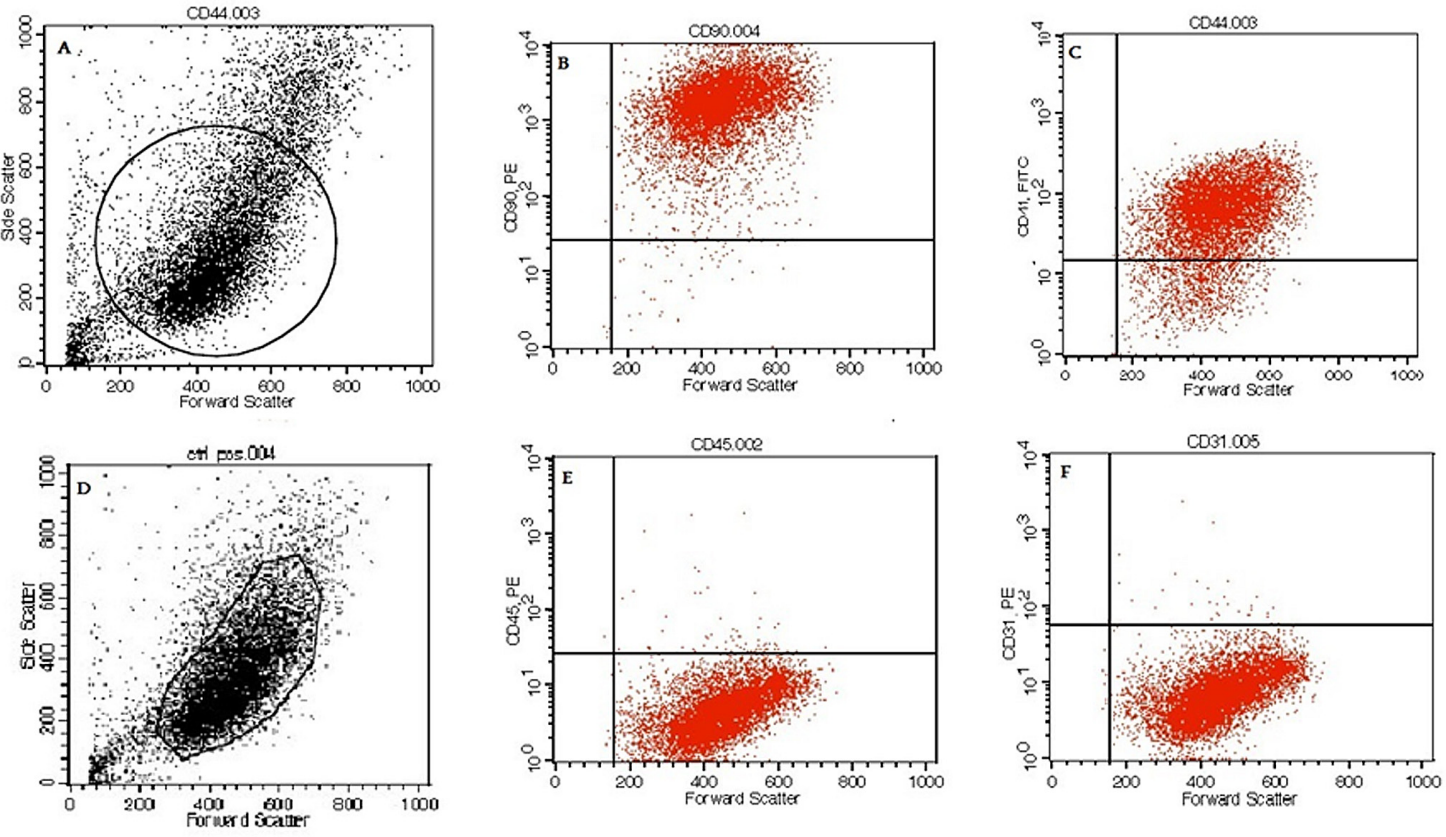
A and D) total population of cells are presented and highlighted population are transferred and tested for different markers, B) CD90-EP positive cells, C) CD44-FITC positive cells, and E) CD45 negative cells and F) C31 negative cells population.

The expressions of surface antigens were detected by flow cytometry using human monoclonal antibodies. Flow cytometry revealed that successfully human amniotic stem cells were isolated based on cell surface marker profiles. All cultures were predominantly positive for CD90 (90%) and CD 44 (80–90%) confirming positive for MSCs markers. In addition, all cells population are checked for CD45 and CD31 expressions and the results showed cells are negative for these markers confirming that they did not express hematopoietic markers (Fig 4).

### Cryopreservation of HAFSCs

1× 10 ^6^ cryopreserved cells for each cryovial in 10% DMSO were thawed and the cell viability was calculated using Trypan blue. The results showed that frozen with 10% DMSO is the best for AFSCs viability.

### Doubling time of hAFSCs

**Fig 5:**
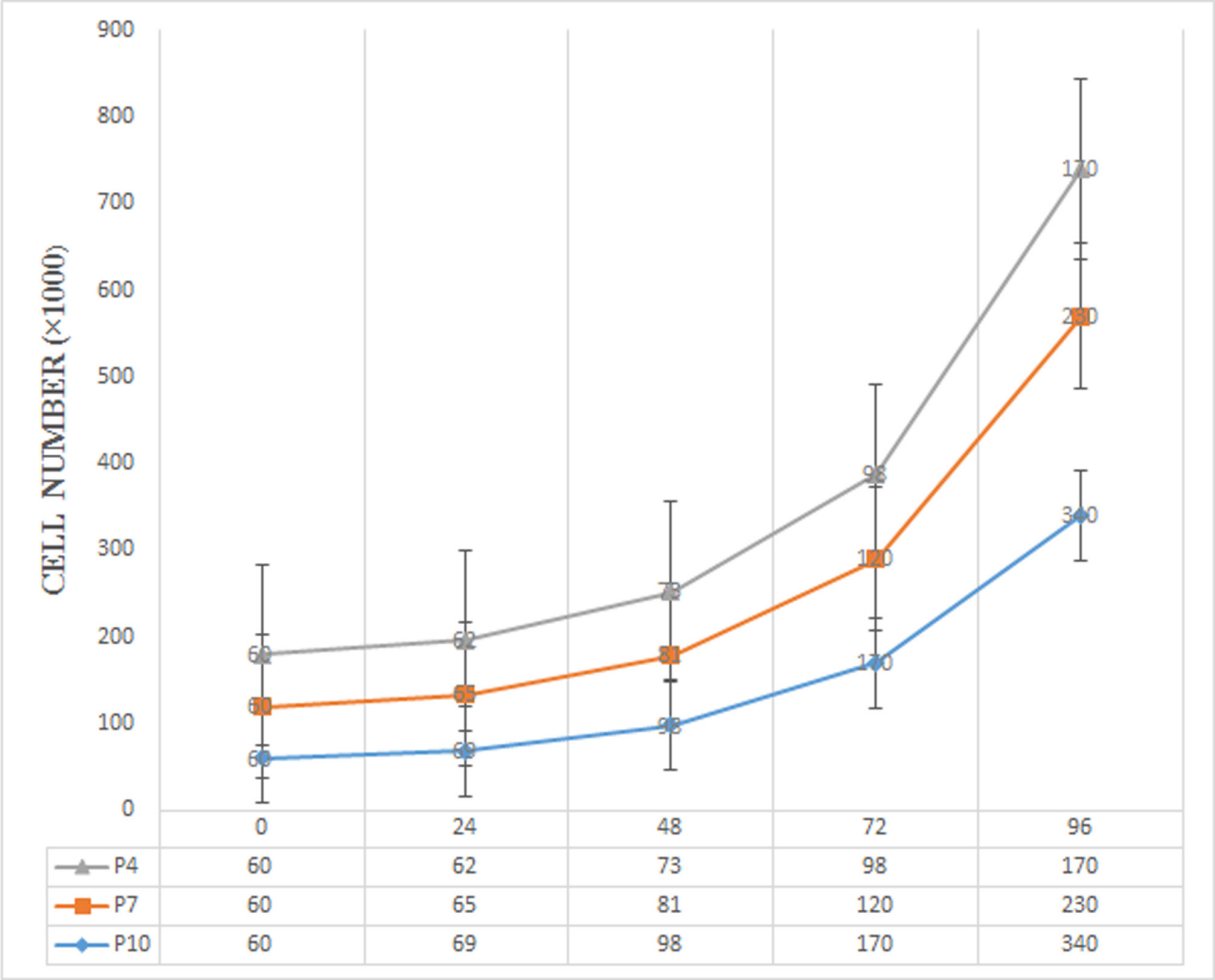
The growth curve of HAFCs in different passages. As shown here, by increasing the time of cell culture, the cell number increased. The growth rate of P4 is faster than P7 and P10. Except the p-value of 0 h vs 24 h, other p-vales were significant.

**Fig 6:**
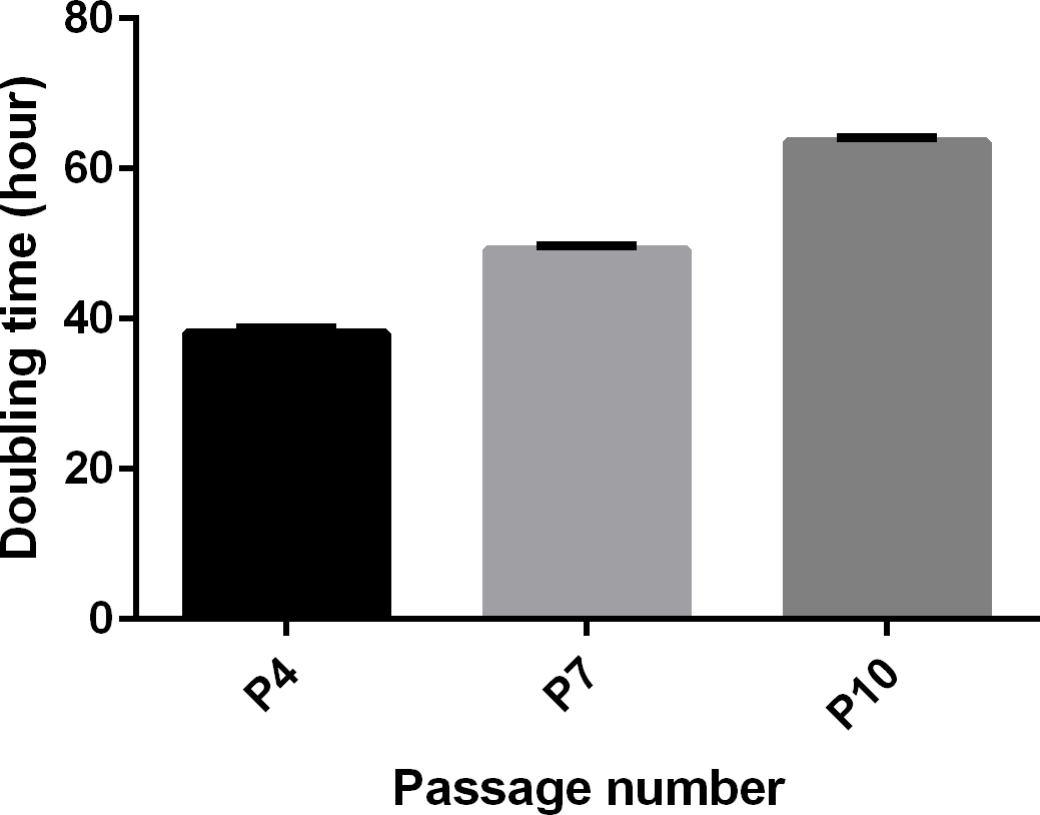
The doubling time of HAFSCs in different passages. As shown in this figure, by increasing the number of passages, the time of doubling time were increased. So, the doubling time related to P10 is significantly more than P7 and P4. All p-value were significant and p-value for P10 vs P7, P10 vs P4 and P7 vs P4 were .000.

The cell growth curves at P4, P7 and P10 were shown in Fig 5. The growth curves were increased by increasing time and the highest doubling time was at P10 after 0,24,48,72 and 96 hour. The double time of hAFSCs was shown in Fig 6.

### Osteogenic and Adipogenic Induction

HAFSCs were able to undergo osteogenic and adipogenic differentiation, as demonstrated by the development of positive staining for Alizarin red and Oil Red after 3 weeks of culture in osteogenic and adipogenic induction medium.

To obtain osteogenic and adipogenic cell population from human amniotic stem cells, we assessed the osteogenic and adipogenic differentiation using Alizarin red and Oil Red. After 3 weeks of culture in osteogenic induction medium, calcium deposited characteristic of osteogenic differentiation in cultures were visualized by positive staining for alizarin red, while the cells were cultured in control medium did not show positivity for alizarin red staining.

**Fig 7:**
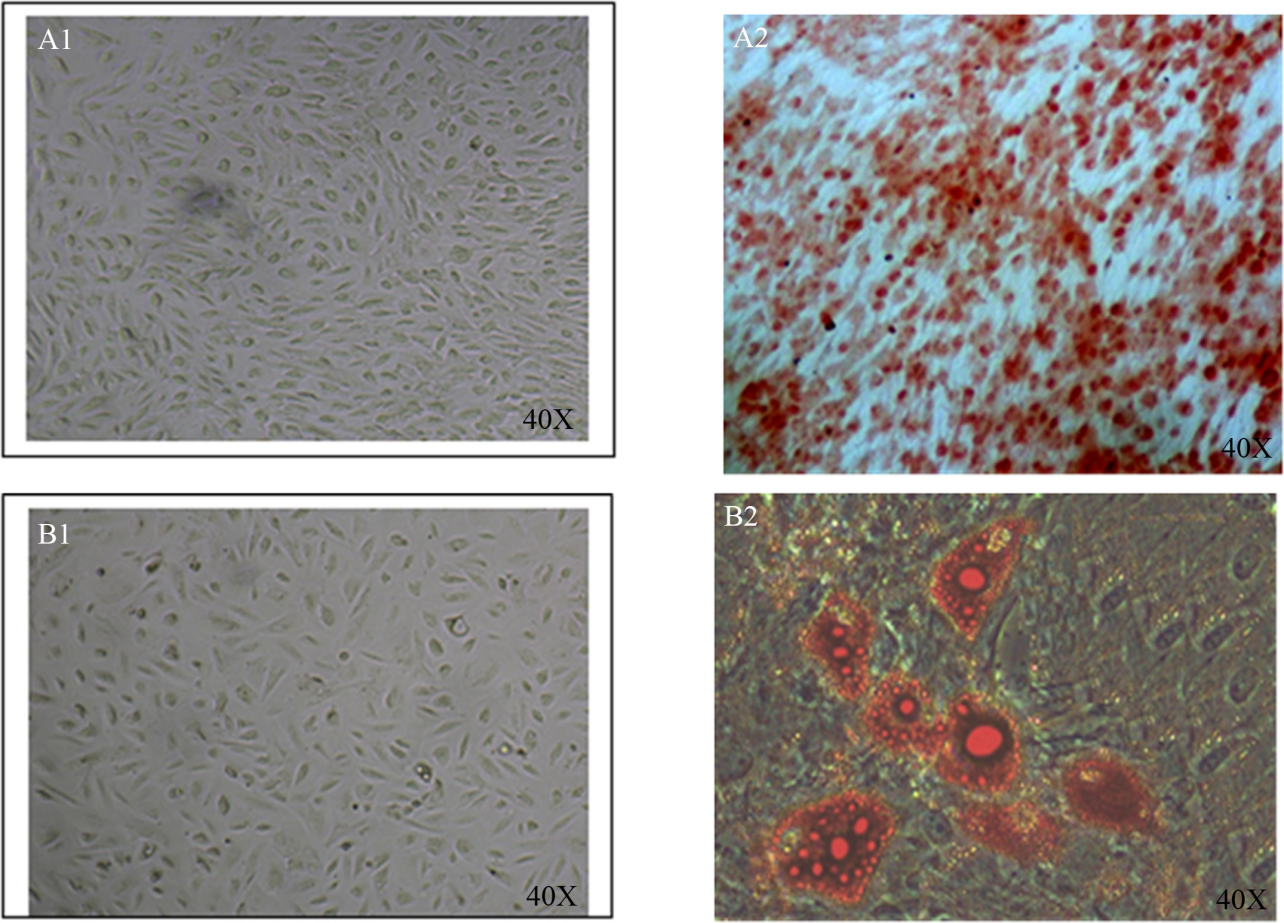
Two lineage differentiation of AFSCs. **A.1, 2** Before and after osteogenic differentiation and cell aggregates (were stained with alizarin red staining).**B1, 2** Before and after differentiation into adipose cells. Arrows show lipid vacuoles generated after adipose differentiation and oil red staining. (40x magnification was used)

To assess adipogenic differentiation, when cells become near-confluent (90%), were cultured for 3 weeks in adipogenic differentiation medium and then, inclusions of neutral lipids in cytoplasmic were stained with Oil Red. The results of staining using Alizarin red and Oil Red (Fig 7) showed successful differentiation to osteogenic and adipogenic cells.

### Network Interaction between NANOG, Oct-4, Adipogenesis and Osteogenesis Based on Common Targets Algorithm

The network constructed by Common Targets algorithm is presented in S1 Fig and its underlying relationships is presented in S1 Table. The hierarchical structure of this network allows us to determine the key targets of NANOG and Oct-4 which have interaction with ossification and osteogenesis in a ranked order. The cellular location and type of different proteins in this network were determined based on Cellular Component definition from Gene Ontology Consortium (http://geneontology.org/) as well as Comparative GO web tool. S2 Fig presents the Common Targets algorithm with determined cellular location. This network highlights the importance of DDK1 (dickkopf WNT signalling pathway inhibitor) as the key ligand which is under the control of (Oct-4/POUSF1) and gas interaction with both ossification and osteogenesis. DDK1 a secreted protein from the dickkopf family and is related to embryonic development through inhibition of the WNT signaling pathway. Increased level of DKK1 in bone marrow plasma is associated with osteolytic bone lesions [47, 48].

**Fig 8:**
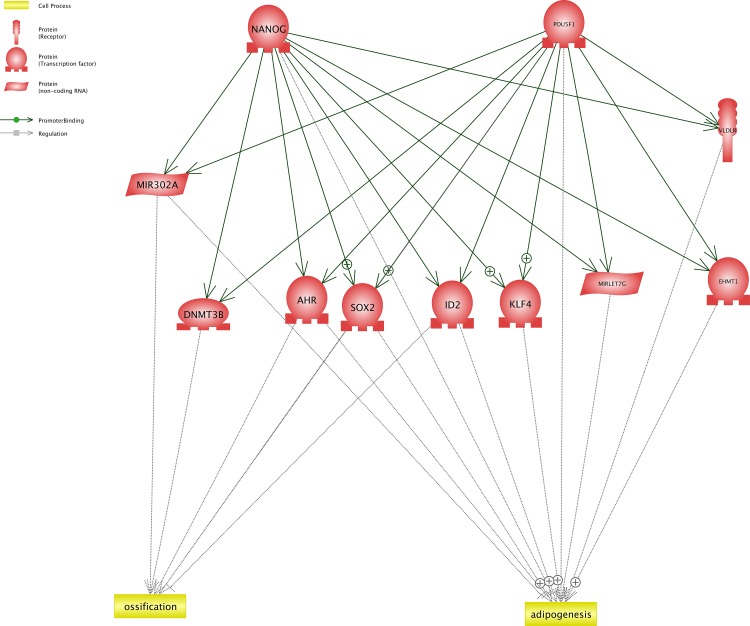
The summarized Common Targets network, keeping only entities which have interactions with POUSF1 and NANOG as well as ossification, and osteogenesis.

**Fig 9:**
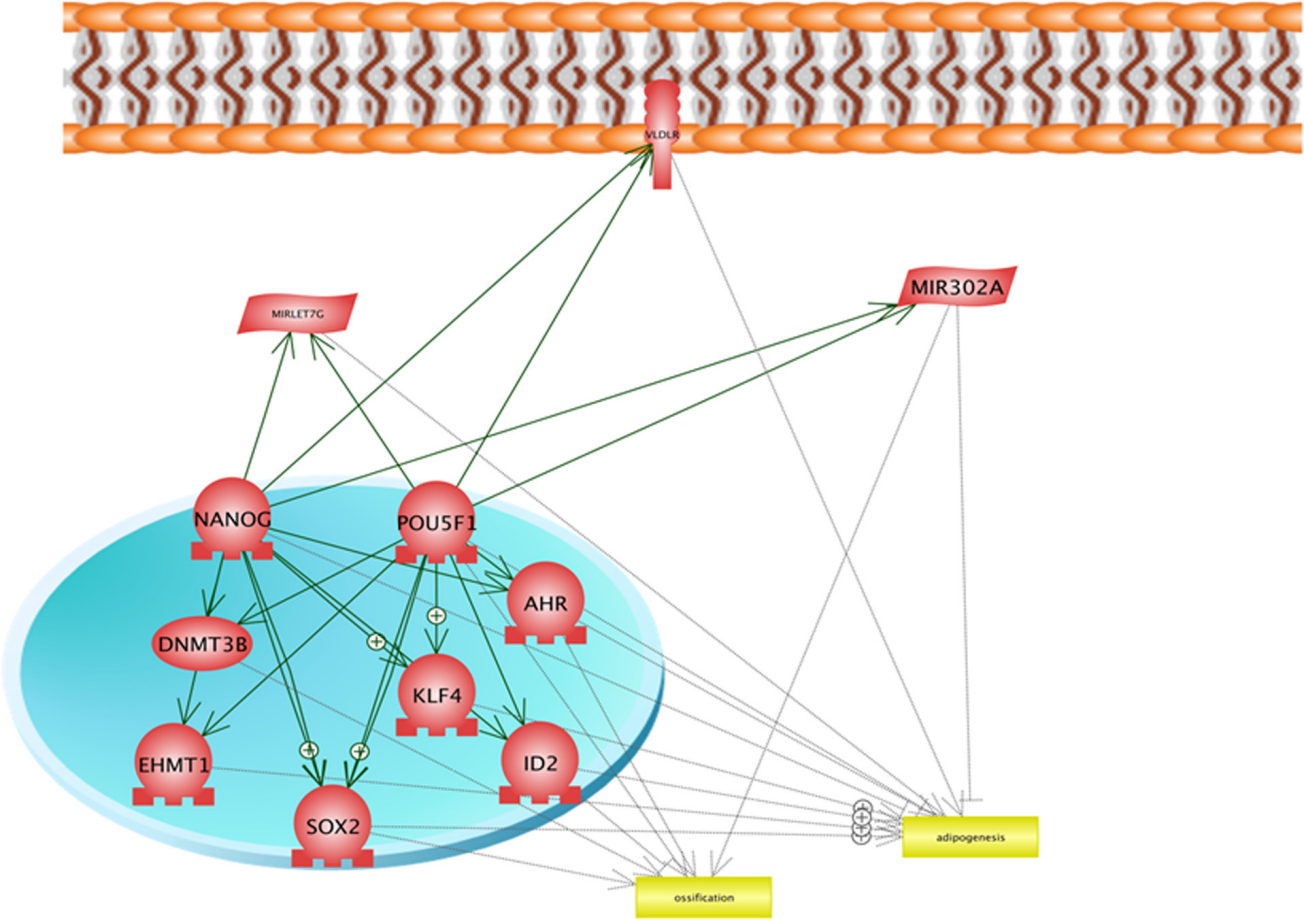
The cellular location of the summarized Common Targets network, keeping only entities which have interactions with POUSF1 and NANOG as well as ossification, and osteogenesis.

The summarised Common Targets network, keeping only entities which have interactions POUSF1 and NANOG as well as ossification, and osteogenesis, are presented in Fig 8. The cellular locations of different components of this network are presented in Fig 9. The constructed network suggests importance of transcription factors including AHR, SOX2, ID2, KLF4 and EMT1 as the targets of NANOG and POUSF1 which has interactions with ossification and/or ossification and adipogenesis. Remarkably, SOX2 and ID2 are under targets of NANOG and POUSF1 and are involved in both ossification and adipogenesis suggesting the key role for this transcription factors in differentiation.

ID2 (inhibitor of DNA binding 2, dominant negative helix-loop-helix protein) belongs to the inhibitor of DNA binding family with a helix-loop-helix (HLH) domain but not a basic domain. This family inhibits the functions of basic helix-loop-helix transcription factors in a dominant-negative manner by suppressing their heterodimerization partners through the HLH domains and are involved in negative regulation of cell differentiation. [Provided by RefSeq, Aug 2011]. Down regulation of ID2 governed by gown regulation of POUSF1 and NANOG can promote both ossification and adipogenesis.

**Fig 10:**
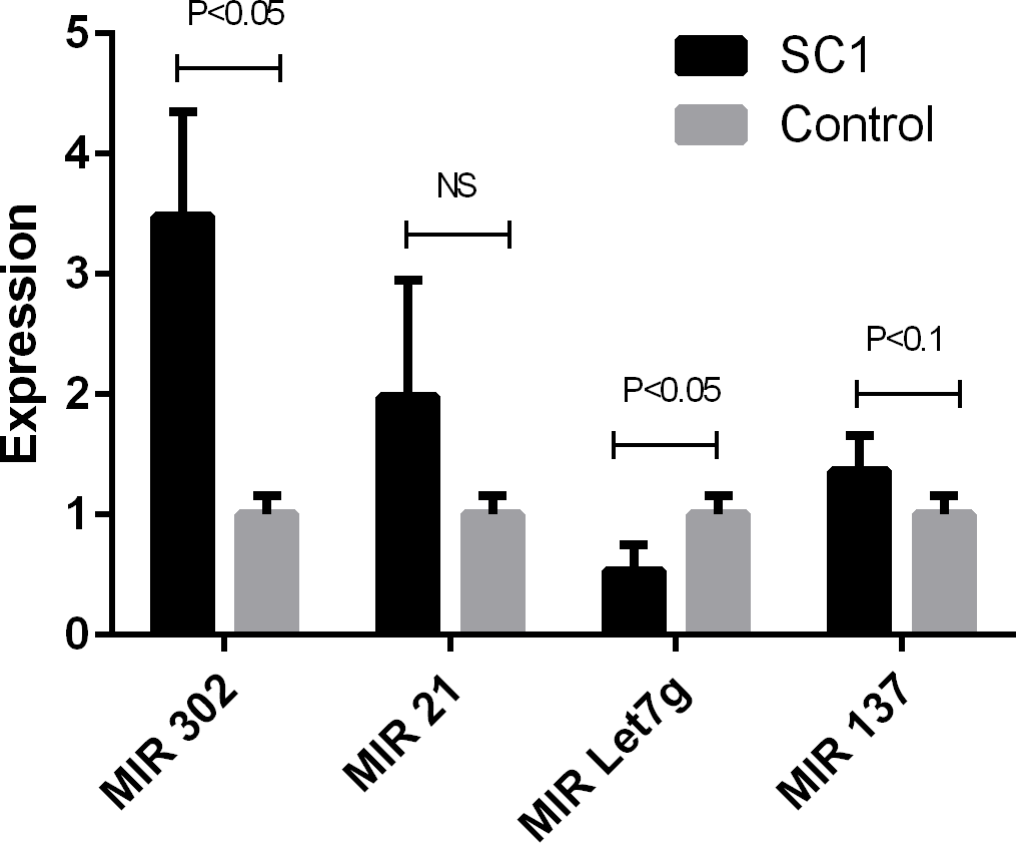
Expression analysis of MIR302A, MIRLet7G, MIR 21, and MR 137 in Human Amniotic Fluid Stem Cells. SC1: Human Amniotic Fluid Stem Cell, Control: Human fibroblasts.

Interestingly, Common Targets algorithm highlighted the possible function of 2 microRNAs including MIR302A and MIRLET7G (Figs 8 and 9) as these microRNAs interact with NANOG and POUSF1 and also are involved in 2 differentiation process of adipogenesis and osteogenesis. MIR302A and MIRLET7G, MIR 21, and MR 137 were selected for validation using qPCR. Result of qPCR analysis is presented in Fig 10. QPCR confirms the result of network analysis as MIR302A showed significant upregulation and MIR Let7g showed significant downregulation in Human Amniotic Fluid Stem Cells (hAFSCs).

### Finding the Upstream Regulators Interacting with NANOG, Oct-4, Adipogenesis and Osteogenesis Based on Common Regulators Algorithm

**Fig 11:**
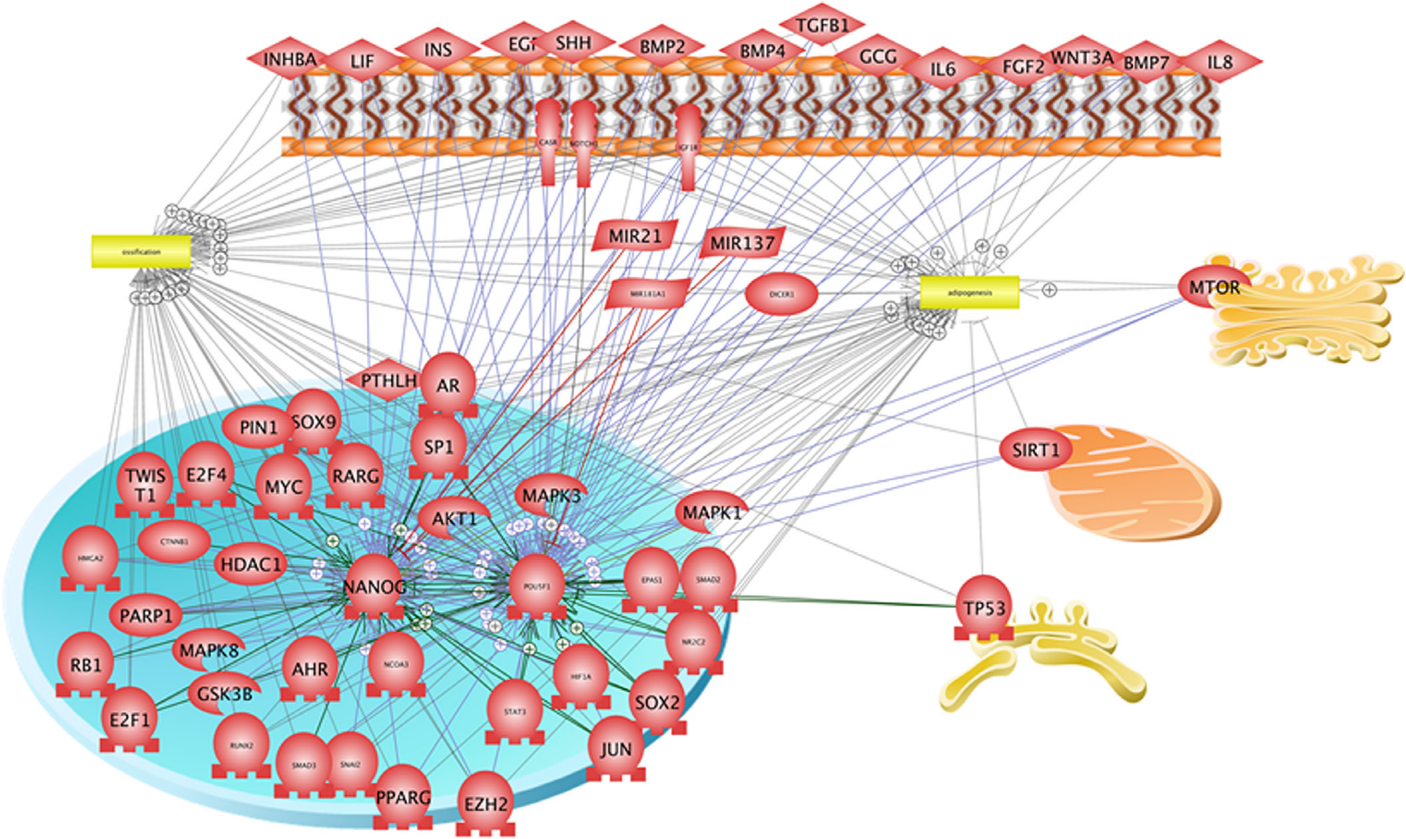
Regulators which can regulate both NANOG, Oct-4, adipogenesis and osteogenesis. The network was constructed based on Common Regulators algorithm and the relationships were extracted by text mining using Pathway Studio 11.0.5 (Elsevier).

The ranked presentation of regulators which regulate both NANOG and Oct-4, adipogenesis and osteogenesis based on Common Regulators algorithm is presented in S3 Fig. The references of its relationships are presented in S2 Table. This network with its cellular location is presented in Fig 11.

Interestingly, this network suggests the key regulatory effects of 3 receptors, including CASR (calcium-sensing receptor), NOTCH, and IGFIR (Fig 11). CASR is a G protein-coupled receptor. CASR is sensitive to even small changes in circulating calcium concentration and translate this information to intracellular signalling pathways to maintain mineral ion homeostasis [49, 50]. The fact that CASR is a central receptor in the regulatory network highlights the important role of calcium concentration in determining the differentiation. NOTCH1 is a transmembrane protein which interacts with golgi organelle and has key function in a range of developmental processes via regulating cell fate decisions This protein is cleaved in the trans-Golgi network, and presented on the cell surface as a heterodimer [51]. Another interesting receptor in the network is IGF1R (insulin-like growth factor 1receptor) which has a key role in transformation events with tyrosine kinase-type activity [52].

### Statistically Significant Subnetwork Generated During Adipogenesis and Osteogenesis in Interaction with NANOG and Oct-4

The gene set enrichment concept was employed to identify and report the key subnetworks. This approach calculates the enrichment/overrepresentation of different subnetworks by an imported list of genes/miRNAs/functions to determine the statistically enriched subnetworks at p≤0.01, based on various statistical measurements, in particular Fisher’s exact test.

S3 Table presents the significant upstream sub-networks (P<0.01) enriched with NANOG, Oct-4, adipogenesis, and osteogenesis according to Gene Set Enrichment where the NANOG and Oct-4 are upstream of the subnetworks. Interestingly, Mir302A which was a hub (central component) in the network constructed by the Common Targets algorithm generate a significant subnetwork with high chance of generation at P<0.001.

Downstream subnetwork discovery, based on gene set enrichment concept, using adipogenesis and osteogenesis, NANOG and Oct-4 as inputs resulted in identification of some central regulatory subnetworks which are presented at S4 Table at p<0.01 which highlighted the key role of transcription factors.

## Discussion

In this study, we isolated hAFSCs and characterized them.Cells presented ability to form colony and maintain for extended passages 15. In addition our cell lines maintained multi-potency after freezing/thawing. These cells were easily isolated from patient’s samples undergoing amniocentesis for routine karyotype screening. Amniocentesis is a moderately gentle modality applied for prenatal and karyotype screening [53]. Our methods may offer an alternative manner of access to fetal stem cells without harm to the fetus itself, which seen in other methods [54].

The pluripotency of hAFSCs has been demonstrated by expressing Oct-4 and NANOG genes and b-actin as housekeeping gene through RT-PCR and real-time PCR (qPCR). NANOG and Oct4 are two transcription factors necessary to sustain the pluripotency and self-renewal of embryonic stem (ES) cells [55, 56]. In this study has been confirmed the expression of pluripotency markers at two different passage of AFSCs.

Over 90% -99% and 85% of hAFSCs were positive for mesenchymal stem cell surface markers CD90 and CD44, respectively which is quite good level of multi-potency of cells. The cells were negative for hematopoietic markers, CD 31 and CD 45 as presented by flow cytometry.

Cells were positive for NANOG and Oct4 as shown by RT-PCR. HAFSCs have been also identified to express pluripotency markers of embryonic stem cells, such as Oct4, NANOG [57, 58] as well as mesenchymal (vimentin, alpha smooth muscle actin α-SMA, N-cadherin) and endothelial (CD144, von Willebrandt factor) [59–63] markers. So, this is the main cause why hAFSCs are well known as an intermediate stage between embryonic and adult stem cells [64, 65].

In this study, we provide a cell bank of undifferentiated hAFSCs which in future may provide a convenient source to patients as therapeutics applications.

HAFSs have potential as precursors to a wide range of differentiated cell linages [66, 67]. Like other studies, we demonstrate the acquisition of lineage-specific functionality by hAFSCs differentiation in vitro toward osteoblasts and adiposity. The multilineage differentiation capability of the cultured hAFSCs was also tested by culturing these cells under specific osteogenic and adipogenic culture conditions. After 3 week induction in this media, hAFSCs had ability to differentiate to adipocytes and osteoblasts as demonstrated by the development of positive staining for Alizarin red and Oil Red. So, it is applicable that seeding hAFSCs in a printed scaffold and exposing to osteogenic medium can form bone after subcutaneous implantation [20].

Furthermore, it is incredible that hAFSCs were shown positive for Oct-4 and NANOG genes, characteristic of the embryonic and adult stem cells, thus proposing their potential pluri- multi-potentiality properties, they are between hESCs and hASCs [22, 68].

The surface marker expression of hAFSCs and their potential for expression of the transcription factor Oct4 proposes that they characterize an intermediate stage between pluripotent ESCs cells [5, 67, 69] and adult stem cells [20].Inconsistency of these results with the anecdotal research outcomes revealing that hAFSCs are holding remarkable abilities for prospective use in the field of regenerative medicine and clinical applications. These cells retain outstanding abilities in therapeutic application compare to hESCs as they are not posed terotoma formation after injection into immunodeficient mice [70].

Network analysis using Common Targets algorithm and Common Regulators algorith provided a better picture of the molecular mechanisms which are possibly involved in ossification and adipogenesis differentiation in relation to NANOG and POUSF1. The constructed network suggests importance of transcription factors including AHR, SOX2, ID2, KLF4 and EMT1 as well as MIR 302A and MIR Let7g. The candidate genes/mechanisms from network analysis provide the required knowledge for future laboratory experiment in stem cell differentiation to osteogenic and adipogenic cells.

It has been reported that Oct4 and Sox2 have binding sites on the promoter region of MIR 302A. MIR 302A is able to repress the key proteins such as cyclin D1and keep stem cell in G1 phase [71]. It has been suggested that MIR 302A is one of the key regulatory components conserved in mammalian pluripotent stem cells [72–74]. It has been documented that MIR 302A has the capability to reprogram human fibroblasts and human skin cancer to pluripotent stem cells [75, 76]. We showed that MIR Let7g is downregulated in hAFSCs for the first time. Less information is available regarding MIR Let7g. MIR Let7g is reported in brain tumor biology, carcinogenesis, and Medulloblastomas [77–79].

In conclusion, our results show the elasticity of hAFSCs and their favorable potential as a multi-potent cell source for regenerative stem cell therapy and their capability to give rise to multiple lineages including osteoblasts and adipocytes. We validated the high expression of MIR 302A and low expression of MIR let7g in hAFSCs by qPCR. These microRNAs were central in gene network of hAFSCs.

## Supporting Information

S1 Fig Network of Human Amniotic Fluid Stem Cells constructed by Common Targets algorithm. (PNG)

S2 Fig Network of Human Amniotic Fluid Stem Cells constructed by Common Targets algorithm with cellular localization. (PNG)

S3 Fig Regulatory network of Human Amniotic Fluid Stem Cells constructed by Common regulators algorithm. (PNG)

S1 Table Underlying relationships of Network of Human Amniotic Fluid Stem Cells constructed by Common Targets algorithm. (CSV)

S2 Table Underlying relationships of Regulatory network of Human Amniotic Fluid Stem Cells constructed by Common regulators algorithm. (CSV)

S3 Table Sub-networks Human Amniotic Fluid Stem Cells enriched with upstream neighbors according to Fisher’s exact test. (XLSX)

S4 Table Sub-networks Human Amniotic Fluid Stem Cells enriched with downstream neighbors according to Fisher’s exact test. (XLSX)
