# Supplementary figures and images for: Isolation, Characterization, Cryopreservation of Human Amniotic Stem Cells and Differentiation to Osteogenic and Adipogenic Cells
Source: PLoS One. 2016 Jul 19;11(7):e0158281. doi: 10.1371/journal.pone.0158281 (PMC4951121; doi:10.1371/journal.pone.0158281)

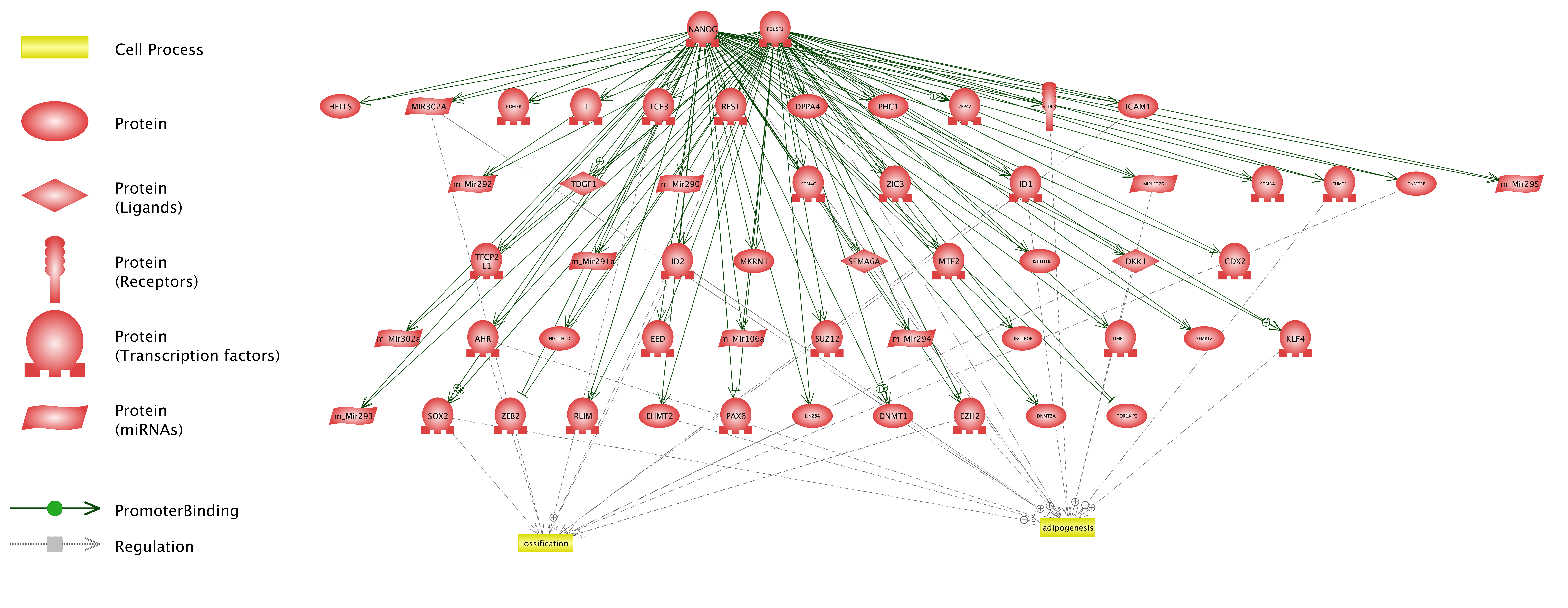

Supplement: S1 Fig — (PNG) [file pone.0158281.s001.png]

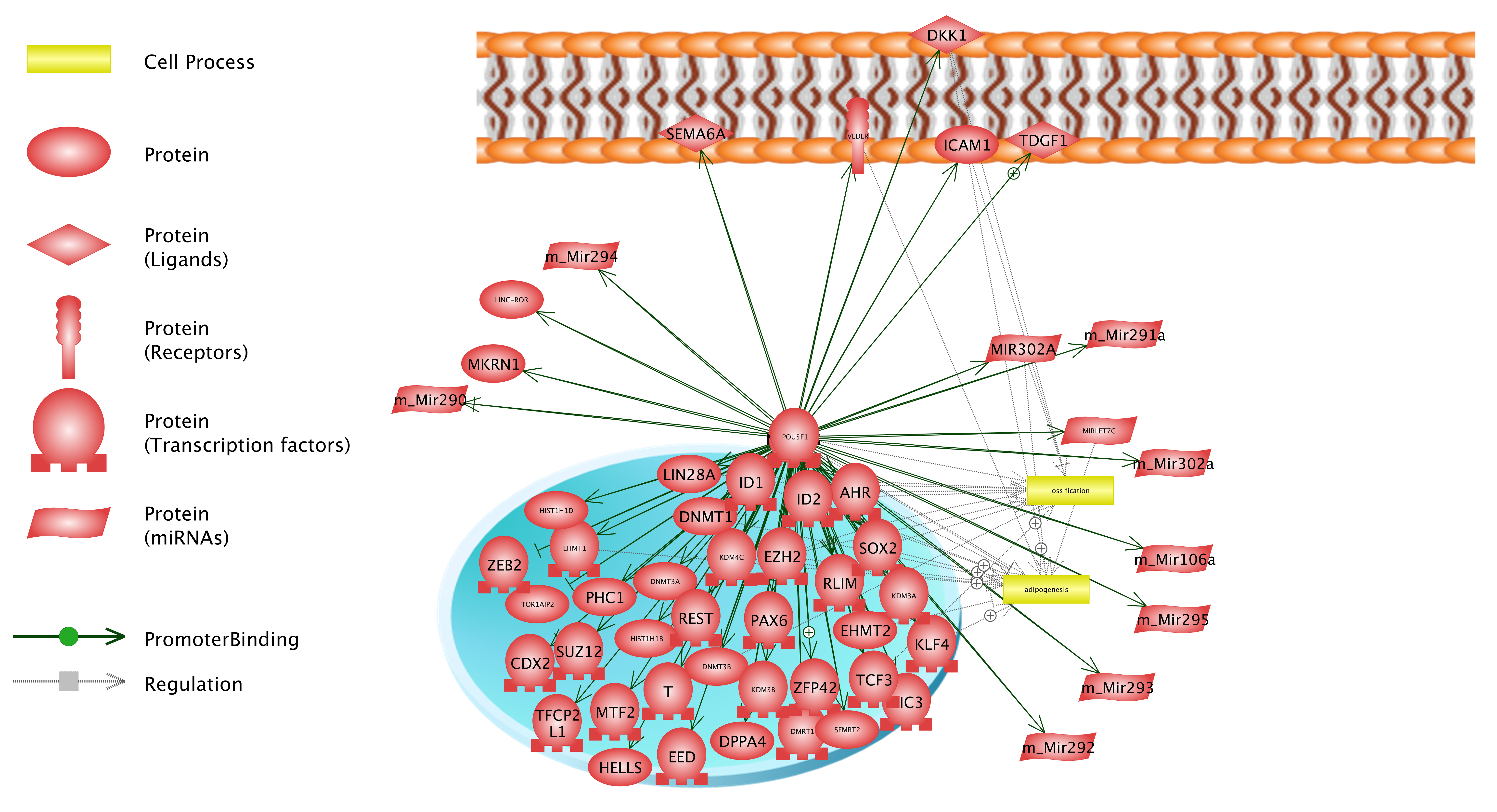

Supplement: S2 Fig — (PNG) [file pone.0158281.s002.png]

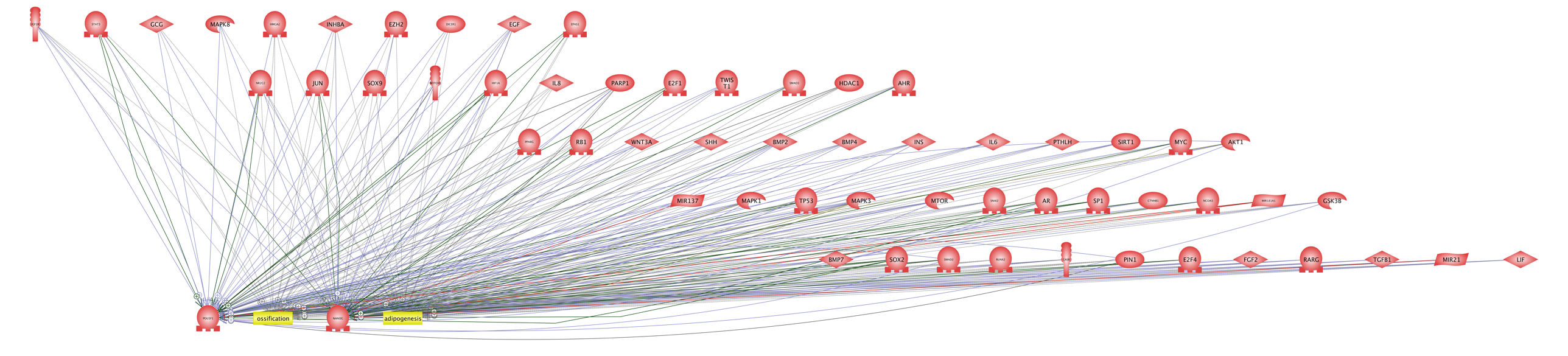

Supplement: S3 Fig — (PNG) [file pone.0158281.s003.png]
